# Spontaneous Preterm Birth Is Associated with Differential Expression of Vaginal Metabolites by Lactobacilli-Dominated Microflora

**DOI:** 10.3389/fphys.2017.00615

**Published:** 2017-08-23

**Authors:** Graham P. Stafford, Jennifer L. Parker, Emmanuel Amabebe, James Kistler, Steven Reynolds, Victoria Stern, Martyn Paley, Dilly O. C. Anumba

**Affiliations:** ^1^Integrated BioSciences Group, School of Clinical Dentistry, University of Sheffield Sheffield, United Kingdom; ^2^Academic Unit of Reproductive and Developmental Medicine, Department of Oncology and Metabolism, University of Sheffield Sheffield, United Kingdom; ^3^Blizard Institute, Barts and The London School of Medicine and Dentistry London, United Kingdom; ^4^Academic Unit of Radiology, Department of Infection, Immunity and Cardiovascular Disease, University of Sheffield Sheffield, United Kingdom

**Keywords:** preterm birth, vaginal microbiome, community state types, metabolites, lactobacilli, lactate, succinate

## Abstract

A major challenge in preventing preterm birth (PTB) is identifying women at greatest risk. This pilot study prospectively examined the differences in vaginal microbiota and metabolite profiles of women who delivered prematurely compared to their term counterparts in a cohort of asymptomatic (studied at 20–22, *n* = 80; and 26–28 weeks, *n* = 41) and symptomatic women (studied at 24–36 weeks, *n* = 37). Using 16S rRNA sequencing, the vaginal microbiota from cervicovaginal fluid samples was characterized into five Community State Types (CST) dominated by *Lactobacillus* spp.: CSTI (*Lactobacillus crispatus*), CSTII (*Lactobacillus gasseri*), CSTIII (*Lactobacillus iners)*, CSTV (*Lactobacillus jensenii*); and mixed anaerobes—CSTIV. This was then related to the vaginal metabolite profile and pH determined by ^1^H-Nuclear Magnetic Resonance spectroscopy and pH indicator paper, respectively. At 20–22 weeks, the term-delivered women (TDW) indicated a proportion of CSTI-dominated microbiota >2-fold higher compared to the preterm-delivered women (PTDW) (40.3 vs. 16.7%, *P* = 0.0002), and a slightly higher proportion at 26–28 weeks (20.7 vs. 16.7%, *P* = 0.03). CSTV was >2-fold higher in the PTDW compared to TDW at 20–22 (22.2 vs. 9.7%, *P* = 0.0002) and 26–28 weeks (25.0 vs. 10.3%, *P* = 0.03). Furthermore, at 26–28 weeks no PTDW had a CSTII-dominated microbiome, in contrast to 28% of TDW (*P* < 0.0001). CSTI-dominated samples showed higher lactate levels than CSTV at 20–22 weeks (*P* < 0.01), and 26–28 weeks (*P* < 0.05), while CSTII-dominated samples indicated raised succinate levels over CSTV at 26–28 weeks (*P* < 0.05). These were supported by Principal coordinates analysis, which revealed strong clustering of metabolites according to CST. In addition, the CSTI-dominated samples had an average pH of 3.8, which was lower than those of CSTII—4.4, and CSTV—4.2 (*P* < 0.05). Elevated vaginal lactate and succinate were associated with predominance of CSTI and II over CSTV in women who delivered at term compared with their preterm counterparts. This suggests that *L. jensenii-*dominance and decreased lactate and/or succinate could increase the risk of PTB, while *L. crispatus/gasseri* may confer some protection against inflammation-associated PTB and highlight the need for further study in this area.

## Introduction

Preterm birth (PTB) is the leading contributor to infant mortality and morbidity globally with associated multi-billion dollar health costs (MacDorman et al., 2007; Goldenberg et al., 2008; Blencowe et al., 2013a,b). While much study has focused on the causes and molecular mechanisms of PTB there are still major challenges in identifying which women who are at risk of PTB. In addition to a history of previous PTB, the increased expression of fetal fibronectin in cervicovaginal fluid and a short cervix on ultrasound are observations associated with risk of premature delivery (DeFranco et al., 2013). However, these tests have limited utility for accurate prediction of PTB, which occurs mostly in women with no apparent risk factors. Indeed, it is estimated that more than 60% of PTBs occur without identifiable cause (Goldenberg et al., 2008). Up to half of all PTBs, however, are associated with evidence of infection or inflammation in gestational tissue (Knox and Hoerner, 1950; Goldenberg et al., 2000).

Thus, in order to understand the mechanisms and prognostic indicators of spontaneous PTB, there is a need to elucidate the role of the vaginal microflora. In recent years, the role of human microbiota in maintaining health and influencing disease has become more prominent (Hajishengallis et al., 2011, 2012) with advances in oral and gut microbiology clarifying healthy and diseased phenotypes that may be manipulated to promote health (Turnbaugh and Gordon, 2009; Cryan and Dinan, 2012; DiGiulio et al., 2015; Xu and Knight, 2015). For instance a link between the composition of the vaginal microbiota and the risk of PTB has been noted (Hillier et al., 1995). An increased risk of PTB is associated with a shift from a health-associated microbiota containing high levels of *Lactobacillus* spp., to a more diverse microbiota containing raised levels of several anaerobic bacteria such as *Gardnerella, Mobiluncus*, and various *Bacteroidetes*, such as *Bacteroides, Prevotella*, and *Porphyromonas* spp. (Fredricks et al., 2005; Hyman et al., 2005; Ravel et al., 2011; Fettweis et al., 2014; DiGiulio et al., 2015) healthy microbiota is also associated with maintenance of a low vaginal fluid pH (3–4.5) by a mechanism that involves host factors as well as the production of lactate by lactobacilli in the normal microflora (Linhares et al., 2011; Witkin et al., 2013; Miller et al., 2016; Smith and Ravel, 2016; Witkin and Linhares, 2016) In addition, lactobacilli produce hydrogen peroxide and secrete various factors (such as bacteriocins and anti-adhesive molecules against *Gardnerella vaginalis)* that suppress growth of competing anaerobic species.

Altered proportions of particular bacterial species within the total microbiota affect the chemical composition of vaginal fluid and epithelial cell behavior (Srinivasan et al., 2015; Vitali et al., 2015). For example lactic acid levels are reduced in relation to changes in the microbiota associated with bacterial vaginosis (BV), while the levels of succinate, which is known to be immunomodulatory (Al-Mushrif et al., 2000), or its potential metabolic by-products (such as gamma-hydroxy-butyrate) are reported to be elevated in some cases of BV (McMillan et al., 2015).

However, in many cases BV is sub-clinical, difficult to diagnose and has prevalence rates that differ by ethnicity, being higher in black women (Fettweis et al., 2014). These differences sometimes make the interpretation of published data difficult due to conflicting results, limiting the identification of suitable predictive markers for PTB. Moreover, the implications of these microbiota-metabolite relationships are yet to be fully explored in relation to spontaneous PTB. Therefore, one aim of current work in the field has been to identify any significant, potentially predictive, differences between the microbiota of women delivering at term and that of women experiencing PTB. The over-arching aim of this study was therefore to determine the variation of the vaginal microbiota composition, and their basic metabolite profiles, during the second trimester of pregnancy and the association of these with eventual term or preterm (<37 weeks) birth outcomes.

Here, we present data characterizing the microbiota of a cohort of women chiefly in their second trimester of pregnancy. We also correlate these findings with differences in the profile of a selection of metabolites, detected by ^1^H-Nuclear Magnetic Resonance spectroscopy (NMR), that may indicate a biological mechanism for the microbiota observations (Amabebe et al., 2016a,b). This is because, it is not the mere presence or distribution of the vaginal microflora, but also their interactions with the host vaginal mucosal surface, resulting in metabolic by-products, that can provide useful functional insight into the pathophysiology of spontaneous PTB, as well as assist in identifying women at greatest risk.

## Materials and methods

These pilot prospective cohort studies were approved by the Yorkshire & Humber (Sheffield) Committee of the National Research Ethics Service (REC Number 13/YH/0167).

### Subject recruitment (microbiota and metabolome samples)

The study participants were recruited via the antenatal clinics and the labor ward assessment unit of the Jessop Wing Maternity Hospital, Sheffield, UK.

**Figure 1:**
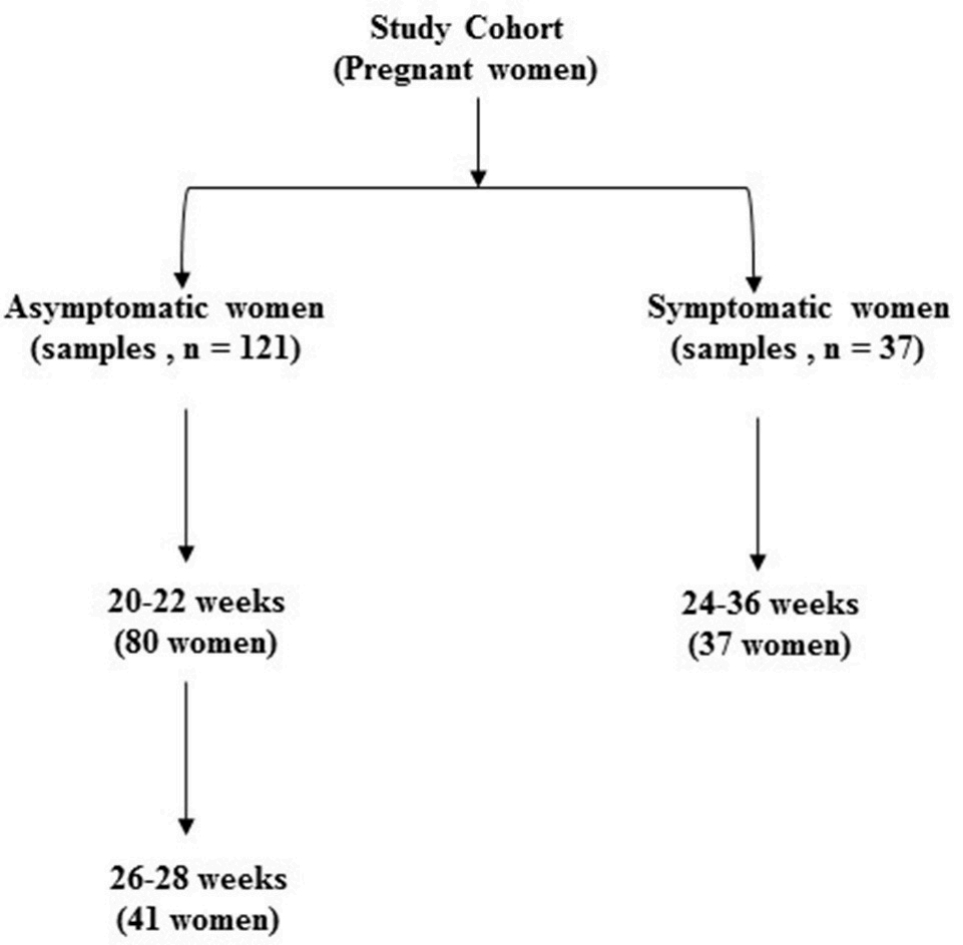
Classification of total study sample population according to presence of symptoms of preterm labor and gestational age at sampling (*n* = 158).

We recruited a cohort of pregnant women comprising those presenting for antenatal care with no symptoms of preterm labor (Asymptomatic, *n* = 121 samples i.e., 20–22, *n* = 80; and 26–28 weeks, *n* = 41) and those presenting to the delivery suite with symptoms of threatened but not established preterm labor (Symptomatic, *n* = 37 samples) (Figure 1). Many of the women in the asymptomatic group (39%) had a previous history of at least one PTB and were therefore at substantial risk of recurrence. Their risk is increased by a factor of 2–6: the earlier the gestation of the previous PTB, or the more the number of previous PTBs, the higher the risk (Hoffman and Bakketeig, 1984; Ekwo et al., 1992; Mercer et al., 1999; Ananth et al., 2006). This asymptomatic group of women were studied between 20 and 22 weeks gestation when they presented for routine antenatal visit. A subset of these women, mainly those with a previous history of PTB (90%), consented to a further assessment at 26–28 weeks, when they re-attended the antenatal clinic. This enabled us to determine whether the microbiota patterns varied in individual women between mid and late second trimester. In addition to this asymptomatic cohort, women attending the labor ward assessment unit with symptoms of threatened, but not established, preterm labor (regular uterine contractions but cervix not dilated beyond 3 cm) between 24 and 36 weeks, consented and provided vaginal swab samples and were included in our “total” cohort. In parallel, we also set out to assess the metabolite profile and vaginal fluid pH of these patients in the same samples.

### Collection of samples

Following written informed consent, clinical research staff obtained two high vaginal swab (HVS) samples from the posterior vaginal fornix with dry polystyrene Dacron swabs (Deltalab Eurotubo 300263, Fisher Scientific, UK) from each woman after the passage of a sterile vaginal (Cusco's) speculum. To obtain adequate amounts of CVF, each swab was rolled in a clockwise direction and moved across the posterior fornix for a minimum of 1 min, the first swab being employed for microbiome studies and the second for metabolite assays. The collected samples were immediately processed or stored at −20°C for up to 3 days pending analysis: one swab was used for DNA extraction and the other for metabolite analysis. In addition, a third HVS was sent for routine clinical microbiological culture and sensitivity tests for BV and urinary tract infections.

During vaginal fluid sample collection, the vaginal pH was also determined from a sample of vaginal discharge obtained by a dry swab, which was then smeared on a narrow range pH paper (Ph-Fix, Macherey-Nagel).

### DNA extraction

Swabs were rinsed in 400 μl of PBS (Sigma) and underwent vortex mixing for 5 min. Two-fifty microliters of swab eluate was transferred to a clean tube and incubated with 1.5 mg/ml lysozyme (sigma) for 1 h at 37°C. DNA was extracted using the QIAamp DNA mini kit column (Qiagen) according to the manufacturer's instructions. Purified DNA was eluted from the QIAamp kit column in 100 μl of ultrapure water and stored at −20°C until further processing.

### 16S rRNA PCR and 454 pyrosequencing

A 500 bp region of the 16S rRNA gene (covering V1–V3) was PCR-amplified from 5 ng of extracted DNA sample using primers comprising universal 16S primers 27F (Frank et al., 2008) and 519R (Lane et al., 1985) along with Roche GS-FLX Titanium Series adapter sequences (A & B) for 454 pyrosequencing. The forward primers incorporate 12 base unique barcode sequences (5′-CCATCTCATCCCTGCGTGTCTCCGACTCAG-NNNNNNNNNNNN-AGAGTTTGATYMTGGCTCAG-3′) to enable pooling of samples in the same sequencing run. The appropriate barcoded A-27F and the B-519R (5′-CCTATCCCCTGTGTGCCTTGGCAGTCTCAG-GWATTACCGCGGCKGCTG-3′) primers were used in PCRs with PHUSION Hi-Fidelity DNA polymerase (Thermo Scientific). For the reaction conditions, there was an initial denaturation step of 30 s at 98°C followed by 25 cycles of 98°C for 10 s, 50°C for 30 s, 72°C for 1 min, and a final extension of 72°C for 10 min. PCR amplicons were initially checked using agarose gel electrophoresis and purified using Ampure magnetic beads according to the manufacturer's instructions. Amplicon quantification, QC, pooling, and unidirectional sequencing of the samples was performed using the Lib-L kit and the Roche 454 GS-FLX + Titanium series sequencer by the DNA sequencing facility (Dr. Shilo Dickens), Department of Biochemistry, Cambridge University, Cambridge, UK.

#### Availability of data and material

Our data has been submitted to the NCBI with SRA accession number SRP065627.

#### Sequence analysis

Analysis of sequences was carried out using the mothur analysis suite v1.34.0 (Schloss et al., 2009) based on the Schloss standard operating procedure (SOP) (Schloss et al., 2011). Sequences were first de-noised using AmpliconNoise algorithm (Quince et al., 2011) as implemented by mother. After de-noising, any sequences that were <400 bp in length and/or had one of the following: >2 mismatches in the primer, >1 mismatch in barcode regions, and homopolymers of >8 bases, were removed from the dataset. The remaining sequences were trimmed to remove the primers and barcodes and aligned to the SILVA 16S rRNA reference alignment (Pruesse et al., 2007). The Uchime algorithm (Edgar et al., 2011) was used to identify and remove chimeric sequences. Diversity of the communities was calculated using Simpson's inverse diversity index (Simpson, 1949) and the total OTU richness of the communities was estimated using Chao 1 (Chao, 1984) and CatchAll (Bunge, 2011). The thetaYC metric (Yue and Clayton, 2005) was used to generate distance matrices in mother, which were visualized as dendograms and Principal Coordinates Analysis (PCoA) plots. PCoA plots were generated in R using the rgl package. Sequences were then classified using a naïve Bayesian classifier within mothur with a bootstrap cutoff of 80% using the Vaginal 16S rDNA Reference Database from Fettweis et al. (2012).

Additionally, sequences were clustered into Operational Taxonomic Units (OTUs) at a sequence dissimilarity distance of 0.03 using an average neighbor algorithm, again using the mothur package. Samples were sub-sampled (again using mothur to equal that of the library with the fewest sequences for subsequent OTU-based analyses (2,000)—an action that in the case of our data did not compromise richness or increase rarefaction) (Supplementary Table S1). A heat map comparing samples based on the relative abundances of species-level taxa (species included comprised at least 1% of the community in at least one sample) was generated using Morpheus (https://software.broadinstitute.org/morpheus/) with hierarchical clustering via a Pearson method according to sample composition.

#### Comparative statistics for CST-level data

Given the non-normal and complex nature of vaginal communities (low diversity index with most samples dominated by only one OTU that is present at >95% of the overall population), there were many counts of zero. Therefore, the method used for the analysis was to create a binary variable based on whether the species made up at least half of the sample (count ≥1,000—and thus assigned a Community State Types, CST). This method was independently selected by health statistics experts at the Statistical Services Unit, University of Sheffield (Director, Sara Hilditch). The proportion of women for whom the species made up at least half of their microbiota was compared between the women who subsequently delivered preterm and those who delivered at term, i.e., the proportions of the dominant CST defining OTUs (CSTs I, II, III, V). Confidence intervals were obtained for the estimated difference in proportions (Laud and Dane, 2014), and a 2-sided *P*-value was calculated, all using proprietary code within the R-package. A significance level of 5% was considered valid. Fisher's exact test was also applied to contingency tables reflecting the % of each sample made up of each CST (I, II, III, V).

As microbiota and metabolite profiles may vary with gestation, we analyzed the data at specific gestational time point ranges. However, in some of our additional analyses when assessing pH and metabolites in relation to CST we also pooled term and preterm samples and included samples collected from the labor ward assessment unit into “total” cohorts since the comparator in that case was CST against metabolite or pH and not gestation at assessment.

### Metabolite sample preparation

A swab-end obtained at the same time and anatomical location as the microbiota swab was suspended in 600 μl of PBS (sigma, Tissue culture grade) and vortexed for 5 min to wash the CVF into solution. The swab was disposed of safely afterwards. The eluate was then centrifuged at 13,000 rpm for 3 min to separate swab particles and any cellular material from the vaginal fluid solution. The supernatant was carefully aspirated into a separate clean 1.5 ml microfuge tube and stored at −80°C until analysis. Prior to ^1^H-NMR analysis, 380 μl of eluate was mixed with 20 μl of deuterium oxide (D_2_O) and transferred into a 5 mm NMR tube (Norell, Marion, NC). With new batch of samples and swabs, several unused (sterile) polystyrene Dacron swabs were also prepared and analyzed identically as background signal controls, taking into account any manufacturer batch variation changes—no changes were noted. To compare metabolite levels between term and preterm pregnancy outcomes and between CST groupings we used *t*-tests (with Welch's correction) and ANOVA (with Tukey's multiple comparisons test), as indicated, since, unlike the OTU abundance data the metabolite data were normally distributed (as tested by a D'Agostino & Pearson omnibus test using the software GraphPad Prism package v7).

### ^1^H-NMR spectroscopy

Using a 9.4T Bruker Avance III NMR spectrometer (Bruker BioSpin GmbH, Karlsruhe, Germany), with 5 mm broadband observe probe, ^1^H-NMR spectra were acquired using the Watergate water suppression pulse sequence (NS = 256, D1 = 5 s, AQ = 1 s, SW = 20.6 ppm, TD = 16,446), for each vaginal sample (Hwang and Shaka, 1995). All ^1^H-NMR experiments were performed at 21°C.

Data was acquired and processed using Bruker Topspin 2.1.6 software to produce a phase and baseline corrected spectrum. In order to assign metabolites to the ^1^H-NMR spectra, 2-D NMR spectra were obtained: ^1^H-^1^H watergate-double quantum filtered correlation spectroscopy (DQFCOSY) – NS = 256, D1 = 0.5 s, AQ = 0.832 × 0.022 s, SW = 9.0 × 9.0 ppm, TD = 6,000 × 160; ^1^H-^13^C presat-heteronuclear single quantum correlation spectroscopy (HSQC) – NS = 1,024, D1 = 1 s, AQ = 0.078 × 0.006 s, SW = 10.0 × 150 ppm, TD = 624 × 180; ^1^H-^13^C presat-heteronuclear multiple bond correlation spectroscopy (HMBC) – NS = 1,024, D1 = 1 s, AQ = 0.128 × 0.005 s, SW = 10.0 × 230 ppm, TD = 1,024 × 200; and ^1^H-^1^H presat-clean total correlation spectroscopy (TOCSY) – NS = 16, D1 = 1.5 s, AQ = 0.284 × 0.071 s, SW = 9.0 × 9.0 ppm, TD = 2,048 × 512. All spectra were referenced to the ^1^H lactate signal at δ = 1.30 ppm. A Representative 1-D ^1^H-NMR spectrum is shown in Supplementary Figure S1.

Identified metabolite signals in the ^1^H-NMR spectra were integrated for peak area (which is proportional to metabolite concentration). To correct for differences in vaginal fluid concentration (variation in the swab sample volumes), each metabolite integral was divided by the total spectrum integral (δ = 0.0–10.0 ppm, excluding the residual water signal between δ = 4.7–5.0 ppm) to provide a normalized integral (N.I.), which is reported throughout this manuscript (Bai et al., 2012; Gajer et al., 2012; Amabebe et al., 2016a,b).

Multivariate analysis (linear and logistic regressions) was performed to determine associations between study metabolites and CST subtypes and delivery outcomes.

## Results

The demographic, pregnancy, and delivery outcome details of study participants are summarized in Table 1 (and Supplementary Table S2). Only 16% of all samples were obtained from smokers and 84% of the women were White. We obtained 158 samples from 133 women (including all women studied before 28 weeks), 26 of whom delivered preterm (37 samples) whilst 107 delivered at term (121 samples); and those symptomatic women studied after 28 weeks but before 36 weeks (*n* = 25).

**Table 1:** Patient level clinical cohort data.

	**Total cohort (*n* = 133)**	**Total term (*n* = 107)**	**Total preterm (*n* = 26)**	**20–22 weeks (*n* = 80)**	**20–22 weeks term (*n* = 62)**	**20–22 weeks preterm (*n* = 18)**	**26–28 weeks total (*n* = 41)**	**26–28 w term (*n* = 29)**	**26–28 weeks preterm (*n* = 12)**
Age (mean, years)	28.8	28.2	31.6	30.1	29.6	32	29.3	28.9	30.2
Prev. PTB (%)	35.3	24.3	80.8	38.8	25.8	83.3	80.5	72.4	100
**ETHNICITY**									
White	82.7	85	73	86.3	91.9	66.6	75.6	75.9	83.3
Black	6	2.8	19.2	7.5	1.6	27.7	9.8	3.4	16.6
Asian	3	3.7	0	2.5	3.2	0	4.9	6.9	0
Mixed	2.3	2.8	0	1.3	1.6	0	2.4	3.4	0
No data	6	5.6	7.7	2.5	1.6	5.5	7.3	10.3	0
Smoker (%)	15.8	15	19.2	12.5	8	27.7	12.2	10.3	16.6
Vaginal pH	N/C	N/C	N/C	4	3.97	4.06	4.1	4.06	4.2
Cervical. length (mm)	N/C	N/C	N/C	37.7	39.2	32	31.3	34	25.45
**CST**									
CSTI	^*^31.8	33.6	^*^28	35	40.3	16.6	19.5	20.7	16.6
CSTII	^*^11.4	11.2	^*^12	11.3	9.7	16.6	19.5	27.6	0
CSTIII	^*^43.2	43.9	^*^40	38.8	38.7	38.8	41.5	34.5	58.3
CSTV	^*^9.8	8.4	^*^16	12.5	9.7	22.2	14.6	10.3	25
Other	^*^3.8	2.8	^*^8	2.5	1.6	5.5	4.9	6.9	0

*N/C, average vaginal pH and cervical length values were not calculated due to different values occurring from samples from different stages of gestation from the same patients. Women studied after 28w (n = 25) are not included in this table (see Supplementary Table S2)*.

* *Calculated with patient 20 (P20) data removed as CST changed from V to III between sampling. N.B. sample level data provided in Supplementary Table S2*.

### Community level microbiota analysis

From our 158 barcoded 16S rRNA gene amplicon samples, we obtained a final dataset of 1,264,109 high quality sequences (of which 10,199 were unique) with a mean length of 442 bases after quality filtering, and alignment to the SILVA reference database, screening of the alignment, and removal of chimeras. This provided a final mean yield of 8,000 sequences per sample for further analysis.

**Figure 2:**
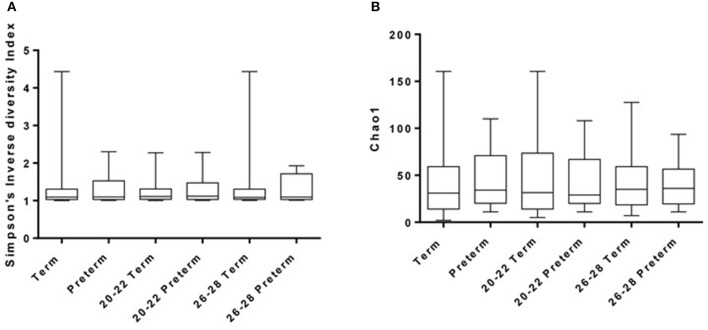
Diversity **(A)** and Richness **(B)** of samples as derived using Inverse-Simpson and Chao1 methods, respectively.

The number of observed OTUs (at a distance of 0.03) per sample after sub-sampling to 2,000 sequences per sample ranged from 1 to 80 (median = 15). The median estimated coverage of the communities according to Good's non-parametric coverage calculator was 99.5% at this sequencing depth. The diversity (Simpson's inverse diversity index) of the communities is shown in Figure 2A, but reveals no significant difference between any of the sample groups i.e. gestational weeks or term versus preterm. Chao1 estimates of total OTU richness for term versus preterm (total, 20–22 weeks and 26–28 weeks) are shown in Figure 2B and were predictably low given the nature of the vaginal microbiota. For example, Chao estimates ranged between 2 and 160 OTUs (with a median of around 30) across all samples (Figure 2).

**Figure 3:**
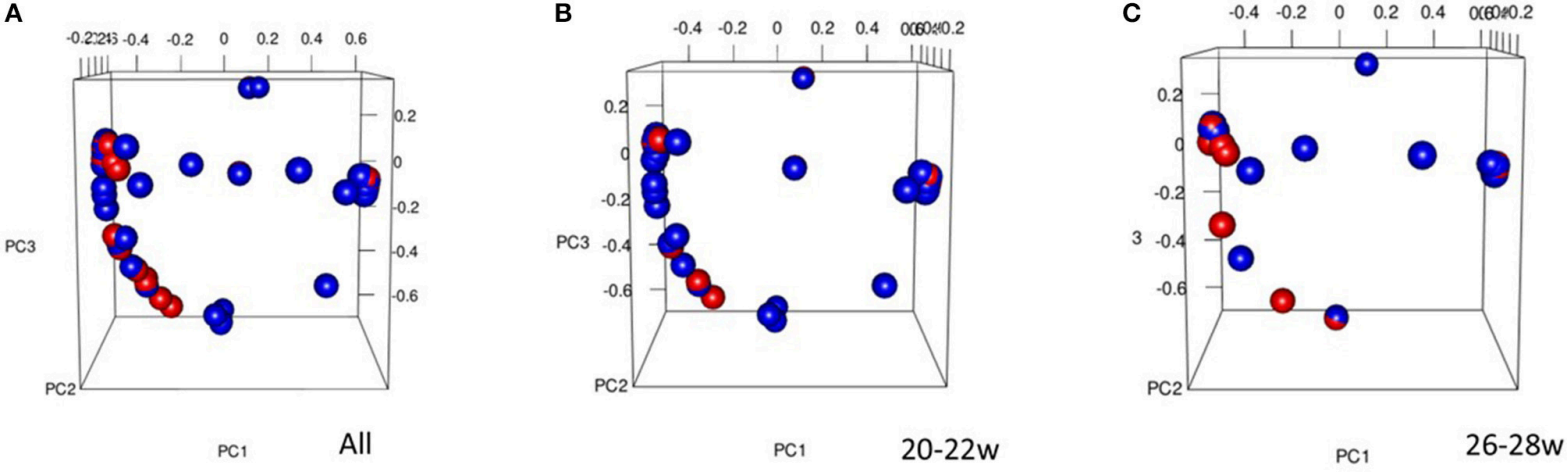
Principal Coordinates Analysis (PCoA) 3D plot of term vs. preterm for All women **(A)** (% proportion of variance attributable to each axis, PC1 = 22.1, PC2 = 11.2, PC3 = 8.5), 20–22 week **(B)**, and 26–28 week **(C)**, Preterm = red, Term = blue. The PCoA was used to explore and visualize any similarities or dissimilarities in relation to delivery outcomes.

Comparison of the overall bacterial community structure of vaginal swab samples from term and preterm women were performed using the thetaYC metric from which a distance matrix was constructed and visualized using PCoA. As shown in Figure 3, there was no significant clustering of samples based on their overall community structure.

#### Assignment of community state type labels

**Figure 4:**
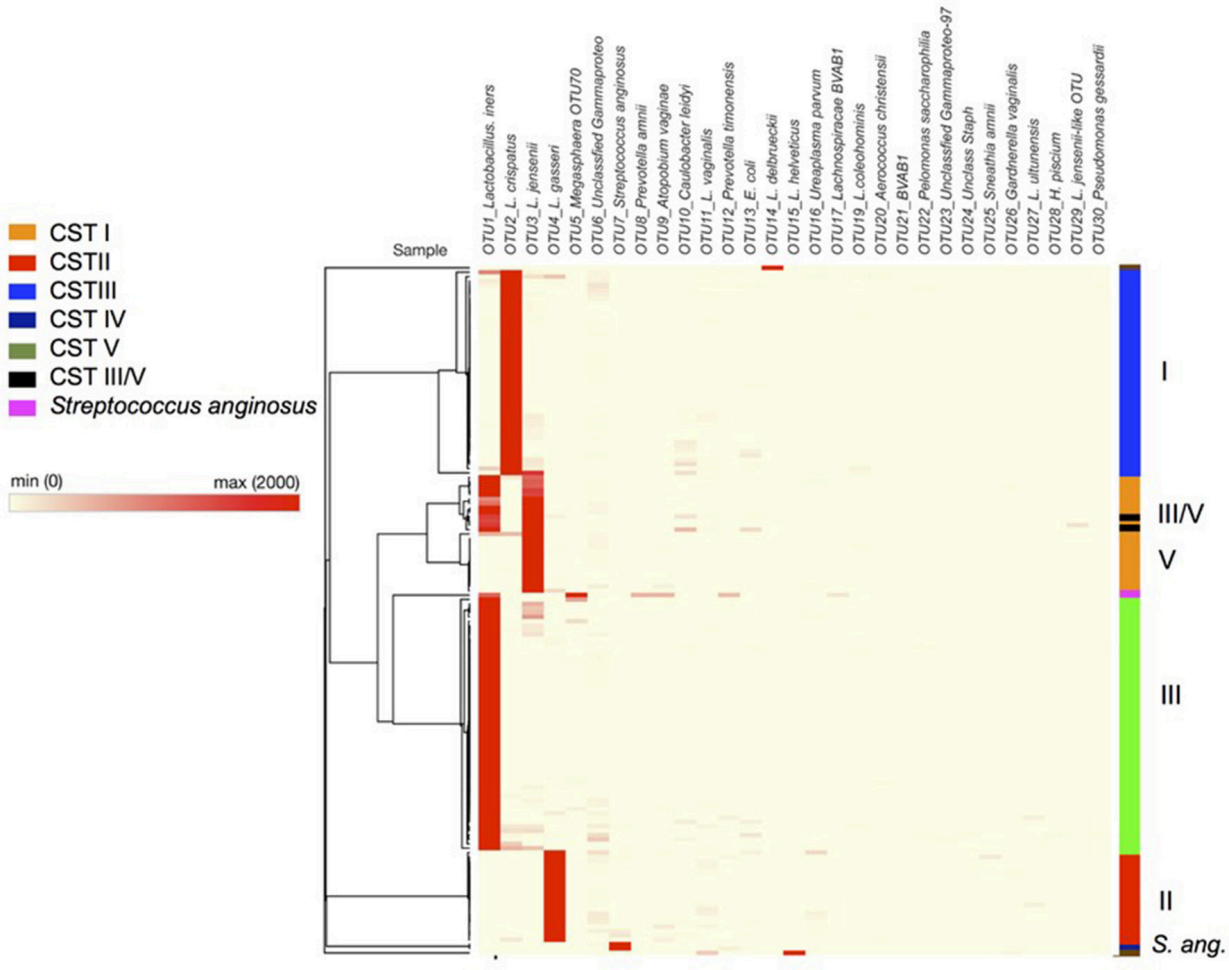
Heatmap of bacterial species composition illustrating hierarchical clustering of data based on OTU level assignment of trimmed data, with community state type highlighted on the left hand side (bar) CSTI (*Lactobacillus crispatus*, blue), CSTII (*L. gasseri*, red), CSTIII (*L. iners*, green), CSTIV (mixed anaerobes, purple), CSTV (*L. jensenii*, orange), CST III/V (50/50 mixed population), *Streptococcus anginosus* (navy blue). Dark red colors indicate high abundance according to the key with each row representing a patient sample. Raw data can be found in Supplementary Table S1 (tabs therein).

**Figure 5:**
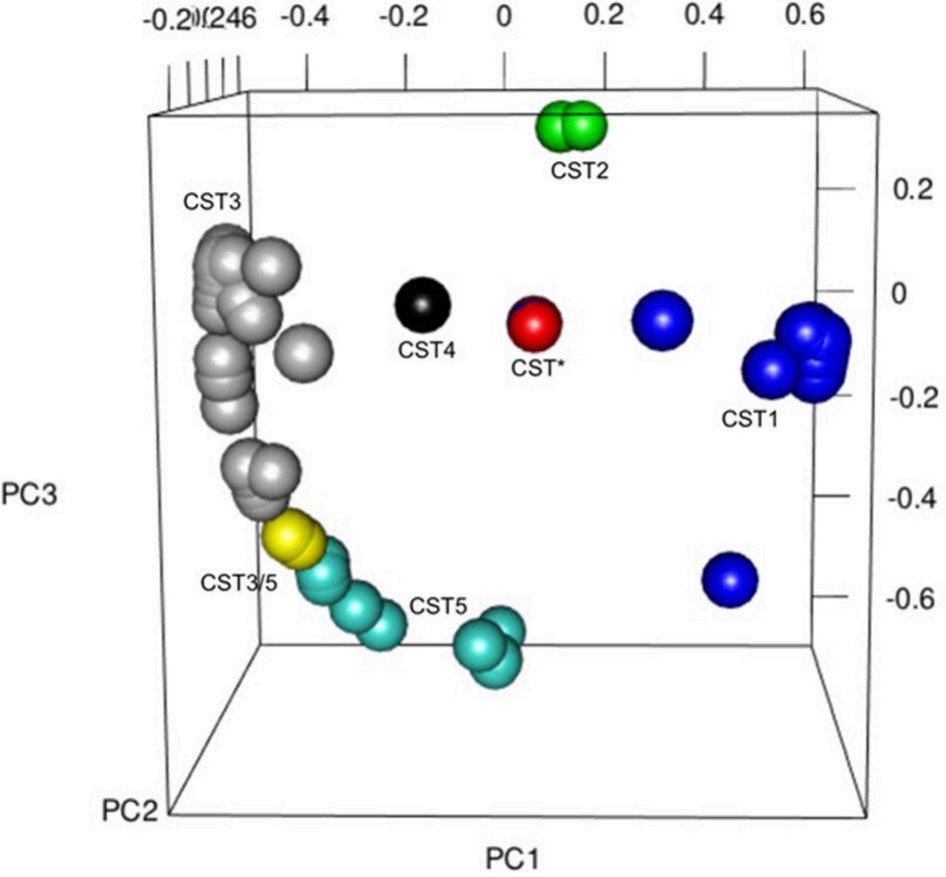
Principal Coordinates Analysis **(**PCoA) 3D plot colored for CST across the whole cohort as follows: CST* = red, CST1 = blue, CST2 = green, CST3 = gray, CST3/5 = yellow, CST4 = black, CST5 = turquoise, Other = brown. The PCoA was used to explore and visualize any similarities or dissimilarities in relation to CST dominance with the % proportion of variance attributable to each axis being PC1 = 22.1, PC2 = 11.2, PC3 = 8.5.

As has been observed in other studies of the vaginal microbiota, analysis of the OTUs in each sample revealed microbial communities that were dominated by *Lactobacillus* spp. (Figure 4). In fact heat-mapping of the OTU abundance in each sample, followed by non-biased hierarchical clustering of the data (using Morpheus) reveals that, like other studies, the communities fall into several distinct clusters that are termed “community state-types” (CSTs) that map onto the abundance of various lactobacilli and other species (Ravel et al., 2011; Gajer et al., 2012). We then assigned CSTs along the lines of previous work (Ravel et al., 2011) with a community dominated by >50% (±5 and 10% higher than next OTU abundance) of OTUs from one *Lactobacillus* species, giving a CST accordingly (CSTI—*Lactobacillus crispatus*, CSTII— *Lactobacillus gasseri*, CSTIII— *Lactobacillus iners*, CSTIV—mixed anaerobes, CSTV—*L. jensenii*). Using this division system we observed that among all the samples combined CSTIII (*L. iners*, 41.14%) was the most abundant, followed by CSTI (*L. crispatus*, 29.75%), CSTII (*L. gasseri*, 12.66%), CSTV (*L. jensenii*, 12.02%), CSTIII/V (*L. iners* and *L. jensenii*, 1.9%), *Streptococcus anginosus* (1.26%), CST* (*Lactobacillus delbrueckii* and *Lactobacillus helveticus* 1.33%), and CSTIV (mixed anaerobes, 0.63%). These assignments were confirmed by repeating the PCoA analyses with samples assigned to CSTs, where, as shown in Figure 5, the CST groups clearly cluster together in the 3D-plots.

CST categorization of the samples confirmed that vaginal microbiota of women at 20–22 weeks who were resampled at 26–28 weeks continued to be dominated by the same species of *Lactobacillus*, with one exception where the woman's CST assignments changed between 20–22 and 26–28 weeks. In the latter case, the microbiota CST changed from ~40:52 (*iners*:*jensenii*) at 20 weeks to ~61:38 (*iners*:*jensenii*) at 27 weeks gestation [Supplementary Table S2, patient 20, P20 (A) and (B)] and showed a mixed lactobacillus population. In addition we found only a small percentage of samples dominated by mixed anaerobes (0.63%, containing, *Prevotella amnii* and *Prevotella timonensis*, alongside *Lachnospiracae* BVAB1 and *Escherichia coli*). Two samples were dominated by *S. anginosus* (a milleri-group *Streptococcus*) (1.3%), while only three of the samples (1.9%) did not show dominance by a single species of *Lactobacillus*, i.e., these samples instead contained approximately equal proportions of *L. iners* (CSTIII) and *L. jensenii* (CSTV) and as a result have been annotated as CSTIII/V.

#### Relationship between CST and outcome

**Figure 6:**
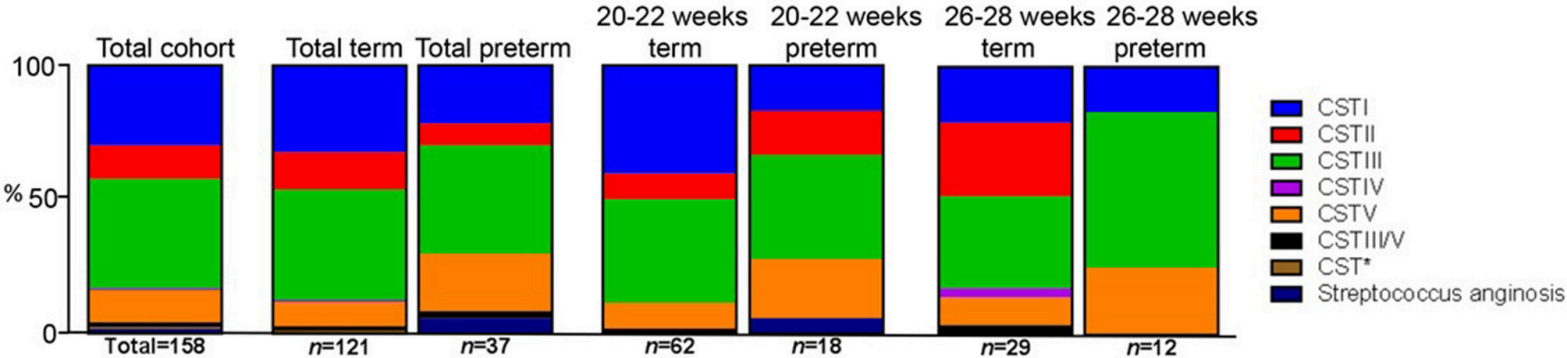
Stacked bar-charts showing CST within total and sub-cohorts showing % of total [number of women in each group (*n*) shown] in each of the CSTs assigned where at least 60% of a sample is composed of a single species of *Lactobacillus* or at least 10% greater than the next most abundant. CSTI (*L. crispatus*, blue), CSTII (*L. gasseri*, red), CSTIII (*L. iners*, green), CSTIV (mixed anaerobes, purple), CSTV (*L. jensenii*, orange), CSTIII/V (50/50 mixed population), Group B (Milleri) *Streptococci* (navy blue) in relation to OTU within normalized trimmed sequencing samples. Total sample population, *n* = 158. CST* (*Lactobacillus delbrueckii* and *L. helveticus*).

When we analyzed CST assignment in relation to birth outcome in our cohorts, we used both a simple Fisher's exact test, as employed by Kindinger et al. (2017) and a much more stringent, alternative test based on the method of Laud and Dane (2014). In terms of data, we observed a greater proportion (>2-fold) of CSTI (*L. crispatus*) dominated microbiota present in the term than the preterm groups at 20–22 weeks (40.32 vs. 16.66% respectively, Fisher's exact test, *P* = 0.0002) and a slightly higher proportion in the 26–28 weeks group (20.69 vs. 16.66% respectively, *P* = 0.03) (Figure 6). Conversely, the proportion of patients' vaginal samples that were categorized as CSTV (*L. jensenii* dominant) was more than 2-fold lower in the term than preterm groups at 20–22 (9.68 and 22.22% respectively, *P* = 0.0002) and 26–28 weeks groups (10.34 and 25% respectively, *P* = 0.03). Also, at 26–28 weeks none of the preterm patient samples were dominated by CSTII (*L. gasseri*) in contrast to 28% of term patients (*P* < 0.0001). When the Laud and Dane method was used, these data were supported at 26–28 weeks, with the lack of CSTII in the patients in the preterm group, *P* = 0.03. Also there was a trend toward a higher proportion of CSTI in the term women compared to their preterm counterparts (*P* = 0.06), again in support of our observations above. Overall, using both statistical methods, there appears to be a link between CSTI—*L. crispatus* and *L. gasseri* and health.

### Relationship between CST and vaginal pH

**Figure 7:**
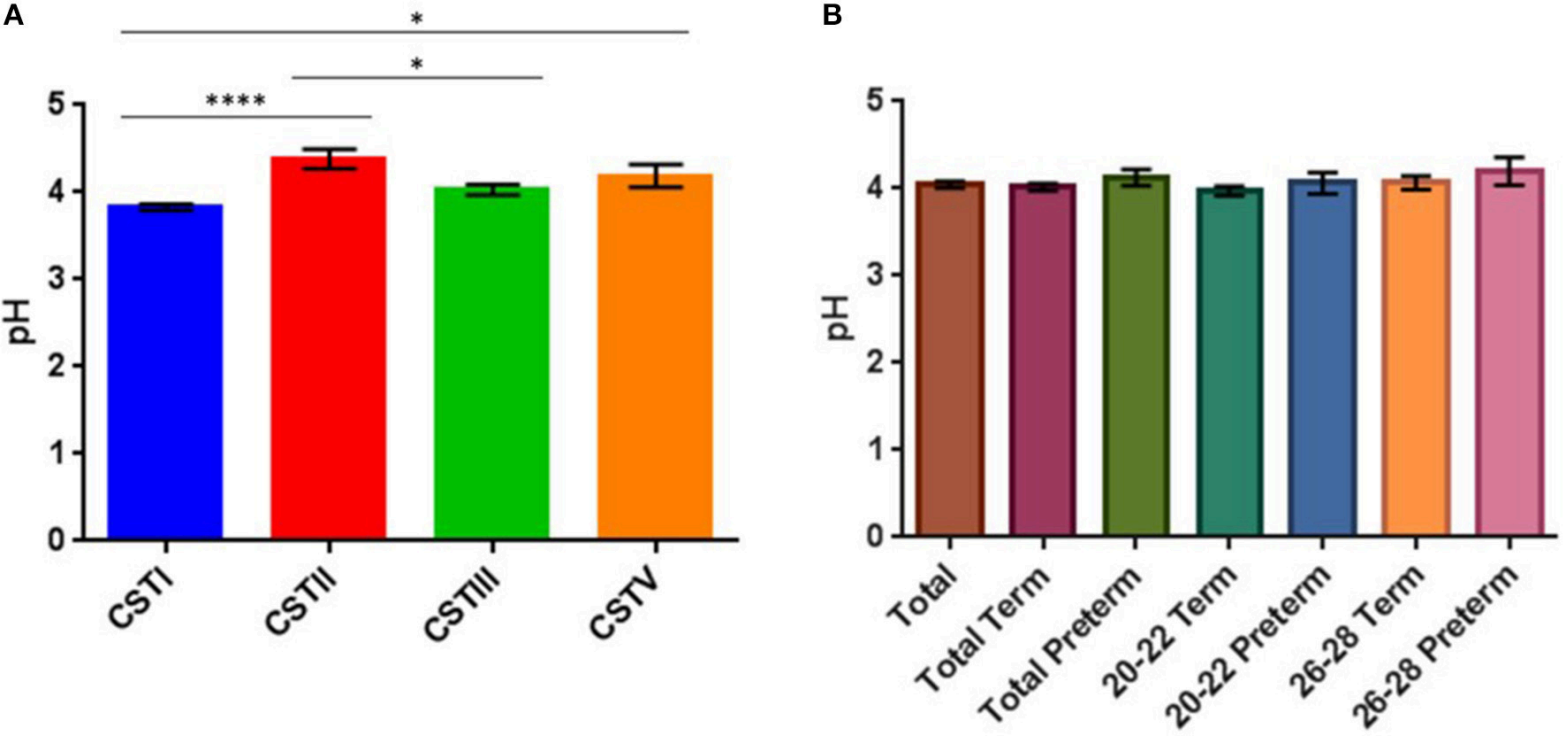
(**A**) Bar plots showing CST in relation to mean vaginal pH (with SEM), (^*^P < 0.05, ^****^P < 0.0001). **(B)** Vaginal pH of total and sub-cohort (no significant differences).

It is well-established that vaginal pH varies in relation to health with a pH of 3–4.5 considered a signature of a healthy vaginal environment and variations upwards a sign of an imbalanced vaginal environment, e.g., onset of BV (Linhares et al., 2011; Smith and Ravel, 2016; Witkin and Linhares, 2016). As a result we measured the vaginal fluid pH of 130 of the 158 samples obtained (N.B. decreased sample population due to lack of patients' consent/data). Our data showed pH-values ranged between 3.6 and 6.1 with a mean value of 4.04 (Supplementary Table S3). We then ascertained whether there was any relationship between pH and CST assignment (Figure 7A). Samples assigned as CSTI (*L. crispatus*) had an average pH of 3.83, with one-way ANOVA analysis revealing that this was significantly lower than samples assigned as CSTII (*L. gasseri*) and CSTV (*L. jensenii*) which had mean pH values of 4.38 and 4.19, respectively (*P* < 0.05). One-way ANOVA analysis also revealed that the mean pH of CSTII samples (*L. gasseri*) was significantly higher than that of CSTIII (*L. iners*), at 4.38 and 4.03, respectively (*P* < 0.05).

### Relationship between outcome and pH

While we see that the mean pH is always lower in the groups that ultimately delivered at term compared to the corresponding preterm groups (Figure 7B) (20–22 weeks: term = 3.97, preterm = 4.06; 26–28 weeks: term = 4.06, preterm = 4.2), this observation was not found to be statistically significant when we performed ANOVA analysis, but may be confirmed with larger sample cohorts.

### Metabolic profiling of our cohort

**Figure 8:**
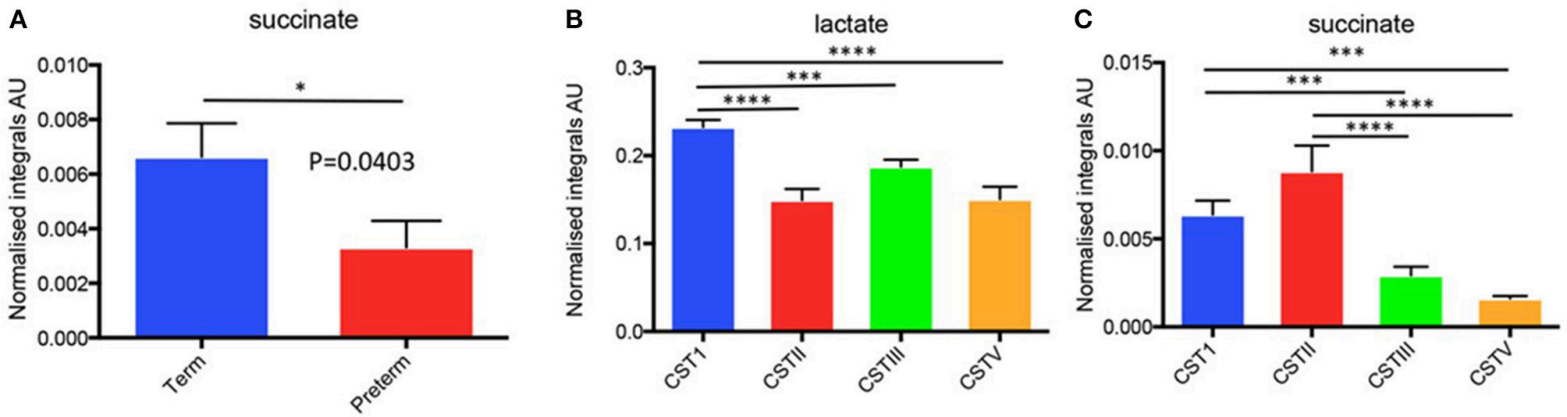
(**A**) Normalized metabolite data for succinate for preterm vs. term in 26–28 week cohort and **(B,C)** Metabolite integrals plotted in relation to CST for the total study cohort (i.e., 20–22 and 26–28 week combined). ^*^P < 0.05, ^***^P < 0.001, ^****^P < 0.0001.

As outlined above we collected parallel samples for microbiota and metabolite data from the same patients under identical conditions. We then performed basic ^1^H-NMR analyses for the six identified metabolites (lactate, glucose, acetate, alanine, succinate, glutamate/glutamine) from these samples, plotting spectra for samples that were then normalized to overall area under the spectrum for each metabolite peak as previously described by our group (Amabebe et al., 2016a). From these data (*n* = 156), we examined the levels of the metabolites in term and preterm women from the same cohorts as for our microbiota studies. Within this data (Supplementary Table S4), we observed that at 26–28 weeks gestation, women who ultimately delivered preterm had significantly lower (about 2-fold) succinate levels compared to the term women (*P* = 0.04, two-tailed *t*-test, Welch correction) (Figure 8A).

**Figure 9:**
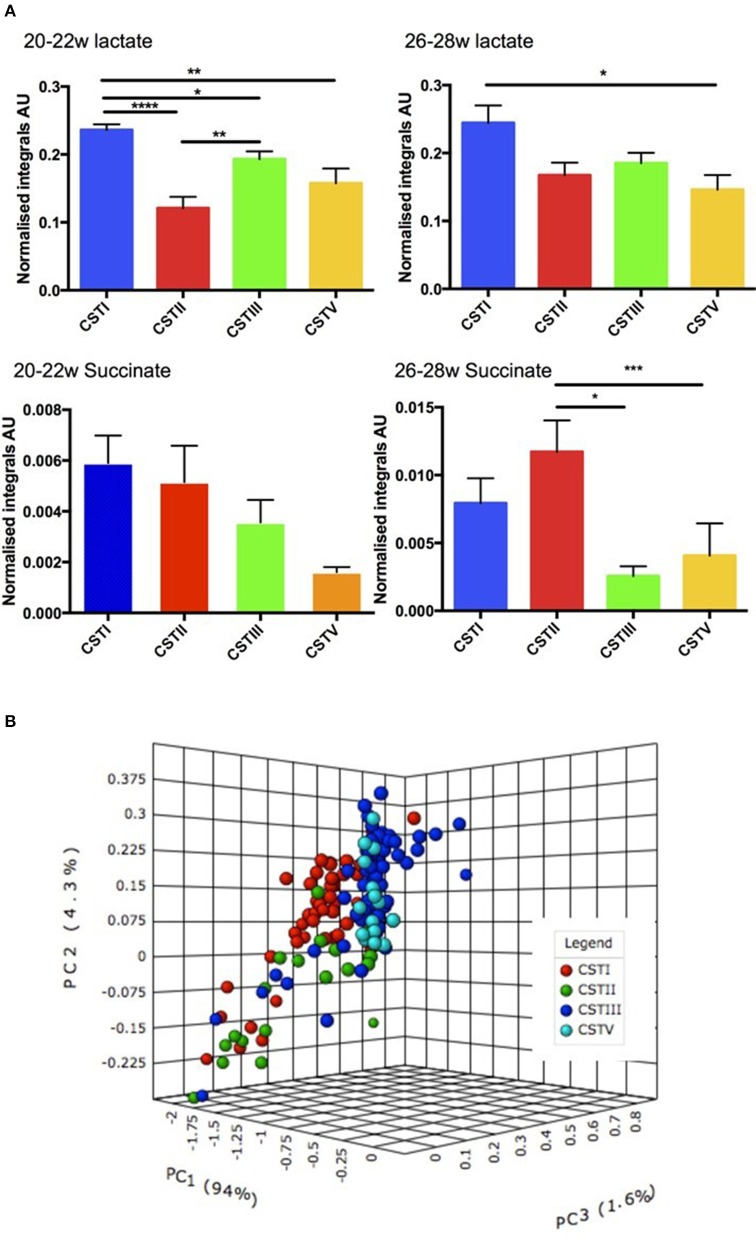
(**A**) Normalized metabolite data for lactate (upper) and succinate (lower) at 26–28 weeks and 20–22 weeks (*AU*, arbitrary units, ^*^P < 0.05, ^**^P < 0.01, ^***^P < 0.001, ^****^P < 0.0001). **(B)** PCoA 3D plot of CST with respect to metabolite levels generated using Metaboanalyst software.

Since we had already identified that the CST profiles of our term and preterm groups differ, observing that *L. jensenii* and *L. crispatus* might be associated with differing outcomes in our cohorts, we then analyzed our metabolite data in relation to CST, using the normalized integrals and One-way ANOVA with Tukeys multiple comparison test applied. As shown in Figures 8B,C, we observed significantly higher lactate levels (1.7-fold higher) in CSTI (*L. crispatus*)-dominated samples compared to those dominated by CSTV (*L. jensenii*) (*P* < 0.01). These differences were replicated for lactate when the groups were analyzed according to gestational age at sampling, with CSTI showing significantly higher lactate than CSTV-dominated samples at both 20–22 weeks (*P* < 0.01), and 26–28 weeks (*P* < 0.05) (Figure 9A). We also observed 4-fold higher succinate integrals in our total cohort between CSTI and CSTV (*P* < 0.001), and although the trend was the same in the subsets categorized by gestational time point of the studies, it did not reach statistical significance. For CSTII (*L. gasseri*), we observed 3- to 5-fold raised succinate levels over CSTV in the total cohort (*P* < 0.0001), at 20–22 weeks (ns) and at 26–28 weeks (*P* < 0.05) (Figure 9A). These associations were supported by Principal Component Analysis of metabolite data from the entire cohort, which revealed strong clustering according to the CSTs (Figure 9B). Multivariate (logistic regression) analysis showed that the other metabolites i.e., glucose, acetate, alanine, glutamate/glutamine had no significant correlations with either CST or delivery outcomes in this unique cohort.

## Discussion

For the first time, we highlight important links between microbial community state-types and targeted metabolite profiles in relation to PTB, thereby highlighting the potential functional and clinical significance of combining these determinations to improve our understanding of the mechanisms of inflammation-associated PTB. We show that community composition (defined as community state type, or CST) seems to differ between women who delivered at term and those who delivered preterm.

This study presents data from vaginal fluid samples of a population of asymptomatic and symptomatic pregnant women studied across mid-trimester, many of who had a previous history of PTB but with a low prevalence of BV or any other genital infection. We analyzed the vaginal microbiota using 16S rRNA gene sequencing while collecting data on pH and metabolite expression levels. Our aim was 3-fold, firstly, to identify any changes in vaginal microbiota early in pregnancy in women who subsequently deliver preterm and to establish baseline information about the microbiota of these cohorts. Secondly, we examined whether any identified changes in microbiota related to wider functional changes in the vaginal microenvironment (i.e., metabolites and pH). Thirdly, we determined if insight could be gleaned from vaginal microbial and metabolite profiles of pregnant women in mid-trimester regarding subsequent PTB.

In relation to the microbiota, it has been established that the vaginal microbiota is relatively simple, i.e., in most cases it is dominated by a single *Lactobacillus* spp. (Ravel et al., 2011; Gajer et al., 2012), and exists in a number of CST classified by dominance of one of four *Lactobacillus* spp., or by a mixed anaerobic population that might indicate dormant or active infection such as BV or aerobic vaginitis (Donders, 2015; Vieira-Baptista et al., 2016). As observed in other studies (Verhelst et al., 2004; Verstraelen et al., 2009; Lamont et al., 2011; Ravel et al., 2011; Gajer et al., 2012; Petricevic et al., 2012; McMillan et al., 2015), the samples from our study population fell into the described CSTs, with the highest abundance of CSTIII (*L. iners*, 41.14%) and CSTI (*L. crispatus*, 29.75%).

Our findings suggest that *L. crispatus*, and potentially *L. gasseri*, might be associated with pregnancies that progress to term and may be “health” promoting, although we acknowledge that larger sample sizes would ideally be needed based on the data gathered here. This is intriguing given the increasing evidence that *L. crispatus*, has a number of phenotypic attributes that might predispose to maintenance of a healthy vaginal microbiota able to out-compete BV-associated bacterial (BVAB) species that are considered risk factors for PTB (Borges et al., 2014; Ghartey et al., 2014). Information on *L. gasseri* is more limited and its potential role in the vaginal microbiota in relation to PTB is unclear at present with suggestions that it is more prevalent in White populations and associated with a more diverse vaginal microbiota (Verstraelen et al., 2009; Hyman et al., 2014).

While *L. crispatus* seems to be associated with health and term delivery, in agreement with others, albeit in different cohort types (Kindinger et al., 2017), our data indicate that in the asymptomatic women studied at 26–28 weeks (90% with a history of PTB), in which ~29% delivered before 36 weeks (12/41), PTB may be associated with *L. jensenii* (CSTV) dominance. We observed that 25% (>2-fold) of women delivering preterm had vaginal microflora dominated by *L. jensenii* compared to 10% of the women that delivered at term. To our knowledge this is the first report of such an observation. However, previously a longitudinal study suggested that in pregnancy *L. jensenii* may be indicative of a more unstable microbiota (Verstraelen et al., 2009), since it was (along with *L. iners*) associated with microbiota that transitioned between CSTs (Jakobsson and Forsum, 2007; Verstraelen et al., 2009). This was also partly borne out in a recent study by Kindinger et al. (2017), who showed an association between *L. iners* and PTB in terms of transitioning microbiota over pregnancy from 16 weeks in a more ethnically diverse cohort than obtained in our second trimester 24–28 weeks study reported here. Contrastingly, *L. crispatus* seemed to be associated with a more stable microbiota, a fact that may influence microbial homeostasis and indicate potential disturbances that are related to progression to infection and PTB (Jakobsson and Forsum, 2007; Donders et al., 2009; Walther-António et al., 2014). Overall this paints a picture of more investigations with larger cohort sizes being key to studies in this area.

Deciphering the role of individual *Lactobacillus* spp. in PTB is daunting given the dearth of knowledge of the microbiology of vaginal lactobacilli. More recently emerging data of genomic sequencing suggest that the genomic complement of *Lactobacillus* spp. differs between the four main *Lactobacillus* spp., potentially indicating adaptations to different environmental conditions (Mendes-Soares et al., 2014). *L. crispatus* has the largest genome of the four main *Lactobacillus* spp. (Witkin and Linhares, 2016), including a plethora of potential bacteriocin and adhesin genes, but how these are expressed, or what their roles are *in vivo* remains unclear (Ojala et al., 2014).

The low abundance of *Bacteroidetes, Gardnerella*, and other BVAB in our dataset was unsurprising given the low prevalence of BV in our cohort. This may be partly due to the narrow ethnic mix—a predominantly white population, but is also borne out of the clinical histories of these women—i.e., no symptomatic BV-clinically and microbiologically verified. This is in contrast to some other studies where the presence of *Gardnerella* and other anaerobic spp. have been shown to be associated with PTB, but were conducted in predominantly African–American women in whom these species are known to be more common (Hillier et al., 1995; Fredricks et al., 2005; Hyman et al., 2005; Ravel et al., 2011; Fettweis et al., 2014). This indicates that any observed association between bacterial species and clinical outcomes in this study may be independent of BV status as all the study participants were tested for BV and those with positive results excluded from the study. However, we acknowledge that our data may be influenced by our primer set, which may have some inherent bias against *Gardnerella* spp. but notably, not other BVAB. However, in contrast to the study of Kindinger et al. (2017), where similar primers were used, we did detect *Gardnerella* in 39 of our 158 samples (albeit at very low levels that in nearly all cases were not indicative of a mixed anaerobe microbiota). In addition, we found only a small percentage of samples dominated by mixed anaerobes (0.63%). This could not be fully explained in the current study because while our primers contained a potential mismatch that could reduce detection of *Gardnerella* spp., they were well suited for detection of a broad variety of *Bacteroidetes* group and *Fusobacterium* spp. as highlighted by their extensive use in assessing anaerobe-rich oral and other rich microbiota samples, including vaginal samples (Lane et al., 1985; Frank et al., 2008; Kistler et al., 2013; Romero et al., 2014). However, clinical microbiological analysis conducted by our hospital's laboratories revealed a low BV prevalence rate (1.9%) in this study population, a fact that explains the low proportion of CSTIV-dominated samples.

Of note is that two samples were dominated by non-CST species of *Lactobacillus*, namely *L. delbrueckii* and *L. helveticus*. The significance of this is currently unclear, but both occurred in women that delivered at term with both species considered potential probiotic species that are present in a significant proportion of healthy female vaginal microbiota (Vasquez et al., 2002; Taverniti and Guglielmetti, 2012). These data also indicate that there are other dominant lactobacilli in the human population that may contribute to the maintenance of a healthy microbiota.

16S rRNA gene sequencing is a powerful tool to establish the composition of the microbial community of clinical (and other) niches in relation to health and disease and to identify potentially associated organisms. However, one drawback of this approach is the inability to examine what the overall functional consequences of the microbiota might be. As a result we utilized a ^1^H-NMR approach to assess the levels of six identified metabolites in the same vaginal fluid samples as used in the gene sequencing experiments. Our data revealed that at 26–28 weeks, women who ultimately delivered preterm had lower succinate levels compared to the term women (Figure 6). This was somewhat unexpected given previous data indicating increases in succinate levels associated with BV and PTB (Spiegel et al., 1980; Ison et al., 1983; Srinivasan et al., 2015). Indeed, data from a similar cohort of women examined by our team (though with a lower prevalence of recurrent PTB), but for which we do not have microbiota data showed a similar but non-significant trend for succinate (Amabebe et al., 2016a), while elevated acetate was predictive of PTB in symptomatic women attending at a late gestation (Amabebe et al., 2016a,b). This indicates that further confirmatory studies are required. However, our cohort contained low levels of BV and it is possible that succinate is linked to the higher levels of *L. crispatus* (CSTI), an organism known to produce succinate (McMillan et al., 2015) compared to other commensal vaginal bacteria (at least *in vitro*) and to express metabolic genes for the production of lactate *in vivo* (Macklaim et al., 2013). Also, compared to our previous study where symptomatic women were examined independently i.e., analyses were based on presentation with symptoms of preterm labor (Amabebe et al., 2016b), the current study analyzed CVF metabolites based on CST dominance, gestational age at sampling, and subsequent delivery outcome. Indeed, when we analyzed metabolite levels in relation to CST designation, we observed significantly higher succinate levels in microbiota dominated by *L. crispatus* (CSTI) and *L. gasseri* (CSTII) compared to *L. jensenii* (CSTV). While intriguing, especially given the immunomodulatory effects of succinate (Al-Mushrif et al., 2000), we must temper this data with the need for a larger cohort of patients in the same groups and a sensitive biochemical assay to assess the concentrations of succinate in vaginal fluid. Nevertheless, in this instance, it could be that the immunomodulatory actions of succinate inhibiting chemotaxis and initiation of an adverse inflammatory state could be protective against inflammation-associated PTB.

We also observed a differential expression of metabolites in relation to the dominant CSTs in the overall study population (i.e., 20–22 and 26–28 weeks combined). There were higher lactate and succinate levels in the CSTI-dominated samples compared to those dominated by CSTV. Similarly, CSTI-dominated samples showed higher lactate levels than CSTV at 20–22 and 26–28 weeks. CSTII-dominated microbiota also indicated raised succinate levels over CSTV in the overall cohort and in women studied at 26–28 weeks only. This was supported by PCoA, which revealed strong clustering of metabolites according to CST in the total population. Taken together, the observed differences in metabolite expression levels in relation to the unique microbiota signature suggests that the dominant Lactobacillus spp. (CST) rather than gestational age at sampling seems to influence specific metabolite profiles.

Our study of vaginal fluid also allowed us to assay the pH of women in our cohorts. While there was a non-significant trend to lower pH-values in the term women, women with high pH-values were not BV positive (Eschenbach et al., 1988; Sheiness et al., 1992). This is in accordance with the low incidence of BV in this study population. However, when the vaginal pH was analyzed in relation to CST, we observed clearly significant differences between CSTI and other CSTs, particularly CSTV-dominated samples (pH: 3.8 vs. 4.2). A potential mechanism for this difference might be the increase in lactate in samples dominated by CSTI (Figure 6), consistent with several studies indicating that *L. crispatus* may decrease vaginal pH *in vivo* by producing high amounts of lactate from glycogen deposits in the vaginal ecosystem (Linhares et al., 2011; Ravel et al., 2011; Witkin et al., 2013; Aldunate et al., 2015).

In this study, we focused on women at risk of PTB in whom the risk of another PTB was high. We did not study any control group of low risk women, matched for gestation, with no symptoms of PTB. We recognize this as a limitation of the present study. It is envisaged that in subsequent studies of sufficiently-powered, larger sample sizes we will explore our observations in unselected populations of women, including those deemed to be at low risk of PTB.

In conclusion, our data present a picture of the vaginal microbiota that tentatively agrees with the potentially beneficial presence of an *L. crispatus*/*gasseri*-dominated microbiota (CSTI/II) over other lactobacilli, particularly the potentially detrimental *L. jensenii* (CSTV). Also, for the first time, we highlight that *L. gasseri* may be associated with vaginal health, albeit in a small, high-risk, largely Caucasian population. Furthermore, our data suggest that CSTs may influence the vaginal microenvironment by altering levels of lactate, succinate and acidity.

Though preliminary, our findings do raise the possibility that further study of the CST Lactobacilli spp. is warranted. Importantly, it highlights the need to focus on the influence of specific lactobacilli-dominated CSTs in larger cohorts of pregnant women including those at low risk for prematurity.

## Ethics statement

This study was carried out in accordance with the recommendations of the Yorkshire and Humber (Sheffield) Committee of the National Research Ethics Service (REC Number 13/YH/0167) with written informed consent from all subjects. All subjects gave written informed consent in accordance with the Declaration of Helsinki. The protocol was approved by the Yorkshire and Humber (Sheffield) Committee of the National Research Ethics Service.

## Author contributions

DA and GS designed the study. JP, GS, EA, DA, VS, MP, SR, and JK all contributed to writing the manuscript with GS, JP, EA, and DA leading the process. JP, JK, and GS processed and analyzed the microbiota data. GS, SR, EA, and MP processed and analyzed the ^1^H-NMR metabolite data. VS and DA collected the clinical swab material that was initially processed by JP and EA. VS and DA collated clinical patient information. All authors read and approved the final manuscript.

### Conflict of interest statement

The authors declare that the research was conducted in the absence of any commercial or financial relationships that could be construed as a potential conflict of interest.

## Supplementary material

Supplementary Figure S1 1-D ^1^H-NMR spectrum of cervicovaginal fluid metabolites. The six metabolites presented are those whose identity could be assigned with high degree of confidence. *ppm*, parts per million.

Supplementary Table S1 Clustering of sequence data into Operational Taxonomic Units (OTU).

Supplementary Table S2 Patients' demographic data.

Supplementary Table S3 Patients' demographics, clinical details and multiplex identifiers (MID).

Supplementary Table S4 Patients' CST classification and normalized metabolite integrals.
