# Supplementary figures and images for: Spontaneous Preterm Birth Is Associated with Differential Expression of Vaginal Metabolites by Lactobacilli-Dominated Microflora
Source: Front Physiol. 2017 Aug 23;8:615. doi: 10.3389/fphys.2017.00615 (PMC5572350; doi:10.3389/fphys.2017.00615)

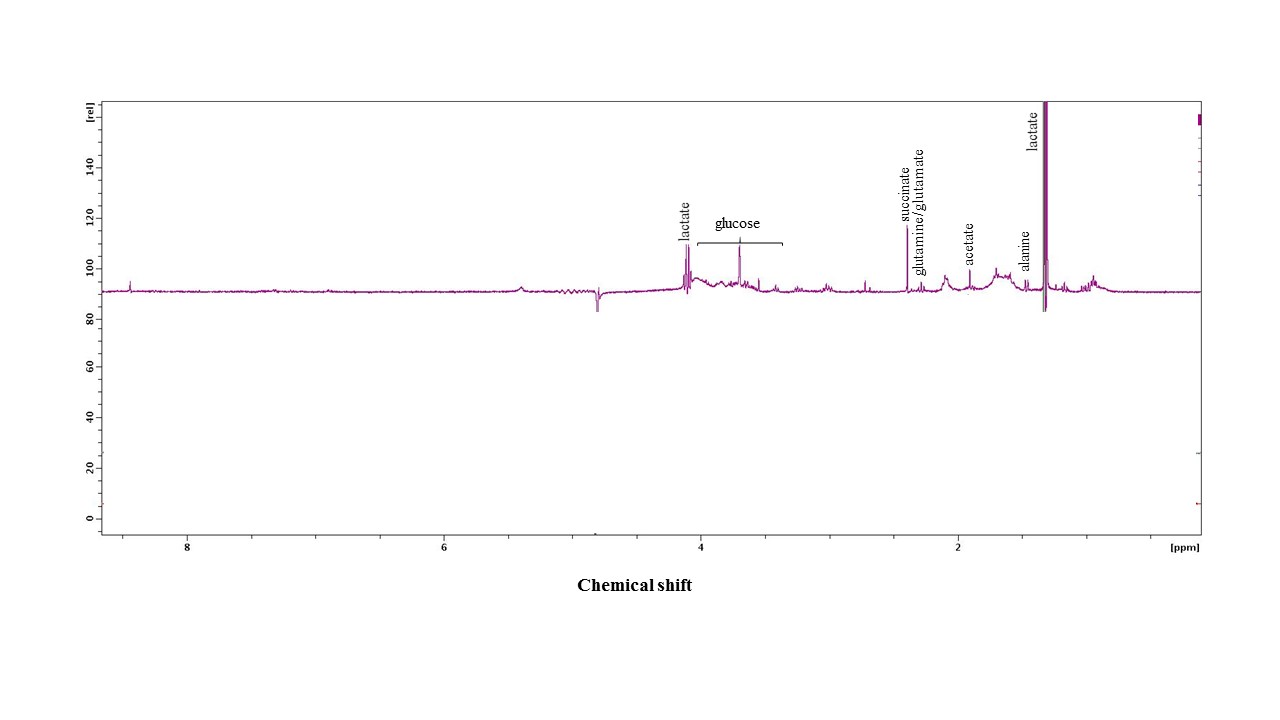

Supplement: Supplementary Figure S1 — 1-D 1H-NMR spectrum of cervicovaginal fluid metabolites. The six metabolites presented are those whose identity could be assigned with high degree of confidence. ppm, parts per million. [file Image1.JPEG]
